# Fabrication of Low-Cost Miniaturized Gas Cells via SLA 3D-Printing for UV-Based Gas Sensors

**DOI:** 10.1021/acsomega.3c09317

**Published:** 2024-02-09

**Authors:** Diandra Nunes Barreto, Rogério Gelamo, Boris Mizaikoff, João Flávio da Silveira Petruci

**Affiliations:** † Institute of Chemistry, Federal University of Uberlândia (UFU), Uberlândia 38400-902, Minas Gerais, Brazil; ‡ Institute of Technological and Exact Sciences, Federal University of Triângulo Mineiro (UFTM), Uberaba 38025-440, Minas Gerais, Brazil; § Institute of Analytical and Bioanalytical Chemistry, Ulm University, 89081 Ulm, Germany; ∥ Hahn-Schickard, 89077 Ulm, Germany

## Abstract

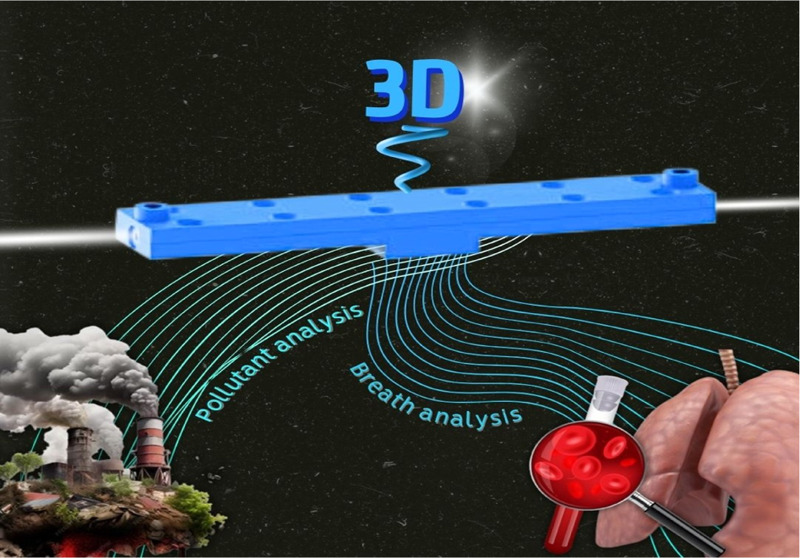

The use of 3D-printing technology for producing optical devices (i.e., mirrors and waveguides) remains challenging, especially in the UV spectral regime. Gas sensors based on absorbance measurements in the UV region are suitable for determining numerous volatile species in a variety of samples and analytical scenarios. The performance of absorbance-based gas sensors is dependent on the ability of the gas cell to propagate radiation across the absorption path length and facilitate interaction between photons and analytes. In this technical note, we present a 3D-printed substrate-integrated hollow waveguide (iHWG) to be used as a miniaturized and ultralightweight gas cell used in UV gas-sensing schemes. The substrates were fabricated via UV stereolithography and polished, and the light-guiding channel was coated with aluminum for UV reflectivity. This procedure resulted in a surface roughness of 11.2 nm for the reflective coating, yielding a radiation attenuation of 2.25 W/cm^2^. The 3D-printed iHWG was coupled to a UV light source and a portable USB-connected spectrometer. The sensing device was applied for the quantification of isoprene and acetone, serving as a proof-of-concept study. Detection limits of 0.22 and 0.03% in air were obtained for acetone and isoprene, respectively, with a nearly instantaneous sensor response. The development of portable, low-cost, and ultralightweight UV optical sensors enables their use in a wide range of scenarios ranging from environmental monitoring to clinical/medical applications.

## 1 Introduction

Three-dimensional (3D) printing has been employed in different fields, from custom-designed prototypes to microfluidics, due to its inherent simplicity and cost-effectiveness. The wide variety of printing techniques and printable materials facilitates the fabrication of complex structures with diverse mechanical and electrical properties. Moreover, the incorporation of substances within the printing materials to modify and optimize their functionalities is increasingly demonstrated.^1^ In the field of analytical chemistry, 3D-printing has been widely used for the fabrication of tailored components integrated into analytical systems and for the fabrication of electrochemical (bio)sensors.^2−5^

Recently, significant attention has been dedicated to the production of active and functional 3D-printed optical devices (i.e., waveguides and mirrors), which have been facilitated by continuing advances in printing technologies. However, the development of 3D-printed optics remains challenging owing to specific requirements including but not limited to (i) optical properties of the printable material (e.g., transparency); (ii) homogeneity of the material to avoid local variations of the refractive index, density, or composition; and (iii) uniformity of the surfaces in terms of flatness and roughness. These factors contribute to the propagation of light, and the performance of the device may be affected by undesirable phenomena such as Rayleigh scattering or light attenuation.^1,6−8^

The identification and quantification of volatile organic compounds (i.e., VOCs) have been performed using optical sensors based on light absorption at a variety of wavelengths. Within the electromagnetic spectrum, the mid-infrared (2.5–25 μm) and the ultraviolet (190–400 nm) spectral ranges are an excellent choice, as a variety of relevant molecules exhibit distinct spectral signatures, enabling a high degree of selectivity. Optical gas sensors are basically composed of a light source, a detector, and a gas cell. Conventionally, the gas cell is an optical device with a well-defined optical path length facilitating quantitative absorption measurements that simultaneously propagate light via multiple reflections and enable the interaction of the gaseous samples with photons.

Among them, substrate-integrated hollow waveguides (iHWGs) have been described by Mizaikoff and collaborators as a modular, miniaturized, and readily adaptable gas cell. The iHWG is a concept tailored for portable and real-time gas detection and has been applied in a variety of scenarios in the MIR, NIR, and UV ranges.^9−12^ Generally, iHWGs are based on a metallic solid-state substrate material (e.g., aluminum) polished up to a mirror-like surface and, in some cases, coated with gold to enhance reflectivity. Although simple and effective, this fabrication method is time-consuming, produces waste due to the subtractive fabrication method, and is relatively costly.^13,14^

The application of 3D-printing approaches to produce optical gas cells is interesting due to the anticipated decrease in weight, cost, and production time. Recently, an iHWG was produced using FDM 3D-printing with ABS filaments, post-treated with acetone, and coated with a gold film. This polyiHWG was employed as a gas cell attached to a QCL/MCT-based system to monitor CO_2_ operating in the mid-infrared spectral range.^13^ However, the challenges related to the fabrication of high-quality 3D-printed mirrors increase with decreasing wavelength of incident light. The surface roughness—expressed as the mean square roughness (Rq)—of the substrate should be related to the radiation wavelength < λ/10 to avoid significant losses in intensity of the propagated radiation. The 3D-printed iHWG employed for the MIR (@10.4 μm) sensing system presented an Rq of 729 nm (λ/14.2). For effective sensing applications using significantly shorter wavelengths, i.e., in the UV, substrates with higher-quality surfaces are essential.

The development of an iHWG for the ultraviolet (UV) range represents a challenging but promising opportunity, especially for gas-sensing applications.^13,15^ The UV spectrum covers a broad spectrum of gaseous molecules that exhibit pronounced absorption characteristics in the 190–400 nm range, which includes specific hydrocarbons, volatile organic compounds, and certain atmospheric pollutants. Accordingly, optical sensors operating in the UV range have the potential to offer fast response times, suitable sensitivity, and minimal cross-reactivity with other substances.^16−18,20^

In this study, we have fabricated a 3D-printed iHWG using stereolithography printing technology, yielding a miniaturized gas cell. By coupling to a UV light source and a portable USB-connected spectrometer, a miniaturized UV absorbance sensor was developed. The performance of the sensing device was evaluated during a proof-of-concept study determining isoprene and acetone vapors in air based on their UV absorption signatures at 228 and 280 nm.

## 2 Experimental Section

### 2.1 Fabrication of the 3D-Printed Miniaturized Gas Cell

An iHWG is composed of two substrates: (i) a bottom part containing the hollow channel and (ii) a top part responsible for light reflection and to seal the cell, ensuring gas containment. Additionally, it features gas inlet and outlet channels that facilitate the introduction of a gas sample into the hollow channel. Herein, both substrates were designed using cad software (Autodesk Inventor 2019) and printed using an SLA 3D printer (Anycubic Photon Mono SE, Shenzhen, China). The acrylic resin was also supplied by Anycubic. Table 1 shows the printing process settings.

**Table 1:** Process Parameters for iHWG Production via 3D Printing

parameter	value
layer height	0.01 mm
bottom layer count	5
bottom lift distance	1 mm
bottom exposure time	30 s
exposure time	10 s
antialiasing level	8

After printing, the substrates were physically polished with a small multipurpose rotary power tool (DREMEL 3000) using resin polishing paste. Surface topography images were acquired using atomic force microscopy (AFM-9600, Shimadzu) and used to evaluate the surface roughness of both substrates before and after the polishing process.

**Figure 1:**
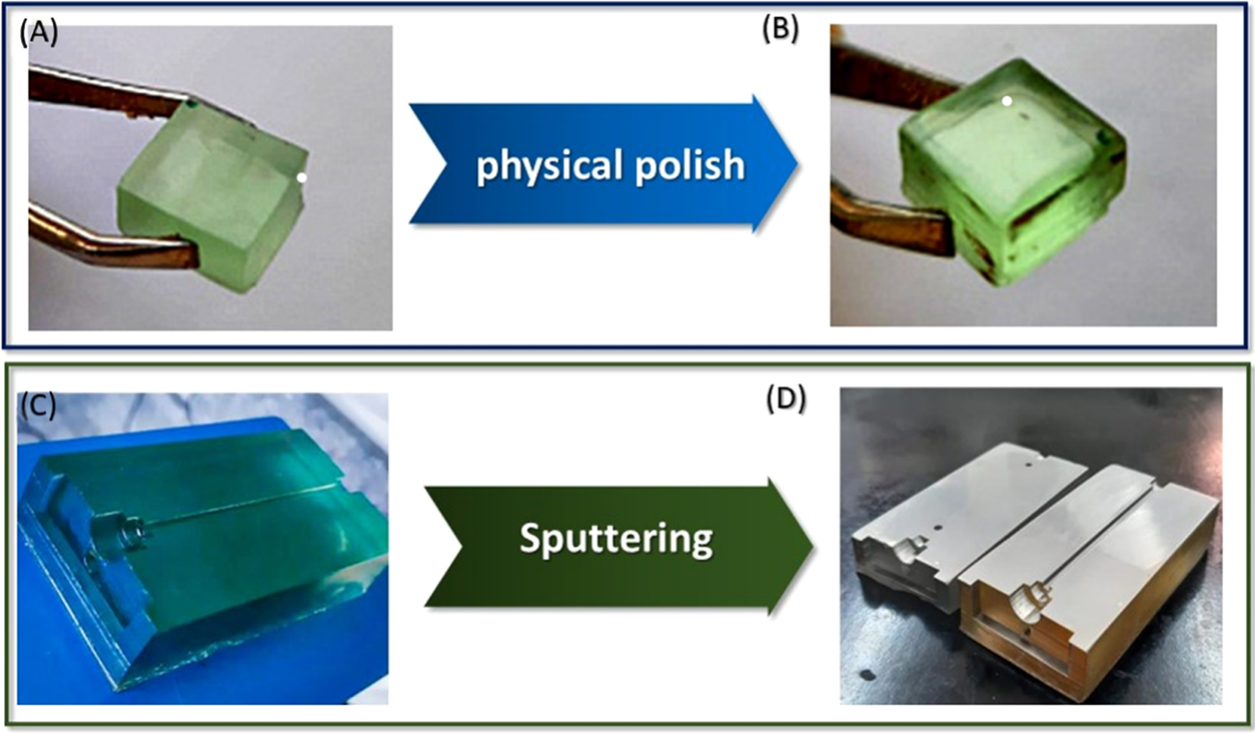
Piece of the printed material (A) before and (B) after physical polishing. Substrates (C) before and (D) after the aluminum film underwent a sputtering process.

Subsequently, to increase the capacity of reflection, the substrates were coated with an aluminum film, which is commonly employed in UV mirror systems.^21^ The effects of the post-treatment processes are illustrated in Figure 1A,B.

The deposition of the reflective aluminum layer was accomplished via DC magnetron sputtering. This process involved evacuation of a vacuum chamber to a pressure of 10^–6^ Torr. Argon gas (99.9% pure, White Martins Co.) was then introduced into the chamber via a mass flow meter until it reached a pressure of 4 × 10^–3^ Torr, with a flow rate of 18 sccm. A DC power supply was employed to generate a current of 140 mA within the magnetron. The aluminum film was deposited under these specified conditions for a duration of 25 min (aluminum target 99.9$ pure purchase from Kurt Lesker Co.). Afterward, to identify the radiation attenuation capabilities of the coated material, radiance tests were conducted on the miniaturized cells using a radiometer (MU-100) fitted with an ultraviolet sensor (250–400 nm).

### 2.2 Vapor Sample Generation and Analysis Protocol

**Figure 2:**
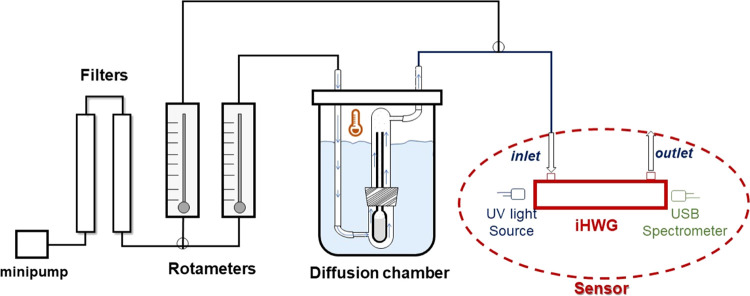
Representation of the standard gas preparation and detection system.

The evaluation of the gas sensor performance was executed by detecting acetone and isoprene (Sigma-Aldrich, St. Louis, MO) vapors generated from liquid solutions of each compound. The procedure employed for generating the standard gas solutions was as follows. First, 800 μL of each solution was transferred into a diffusion tube with dimensions of 10.0 cm × 2.0 cm (length × diameter), as described by Cardoso et al.^22^ The tube was placed into a diffusion chamber kept to a temperature next to the boiling point of each analyte (i.e., 50 °C for acetone and 30 °C for isoprene) using a thermostatic water bath. Subsequently, a 50 mL min^–1^ flow of purified air supplied by a 5 V minipump (RS-385) was used as a carrier gas, and the acetone/isoprene vapor was injected into the UV-iHWG detection system. Before each analysis, the entire system was purged with pure air at a constant flow rate of 500 mL min^–1^ for 5 min. The air was purified by two columns containing KI and silica and regulated by a valve and a primary airflow calibrator (Gilian Gilibrator-2, Sensidyne, Florida). The concentration of each analyte in the generated vapor was confirmed by GC-FID. The experimental setup is shown schematically in Figure 2.

The gas sensor system was based on a deuterium lamp serving as the UV radiation source (Avantes, AvaLight-D(H)-S). The UV light was injected at one end of the 3D-printed iHWG, while a portable USB-connected spectrophotometer (USB 4000, Ocean Optics), used to acquire transmittance and absorbance values, was attached to the opposite side using optical fibers. The data acquisition was conducted using Spectra Suite software with an integration time of 250 ms and 16 scans. This setup allowed for dynamic and rapid responses to changes in the acetone and isoprene concentrations. For comparison purposes, the samples were also analyzed by gas chromatography with flame ionization detection (GC-FID) via a direct injection. The results obtained from both methods were subsequently compared and evaluated.

## 3 Results and Discussion

### 3.1 Surface Evaluation

**Figure 3:**
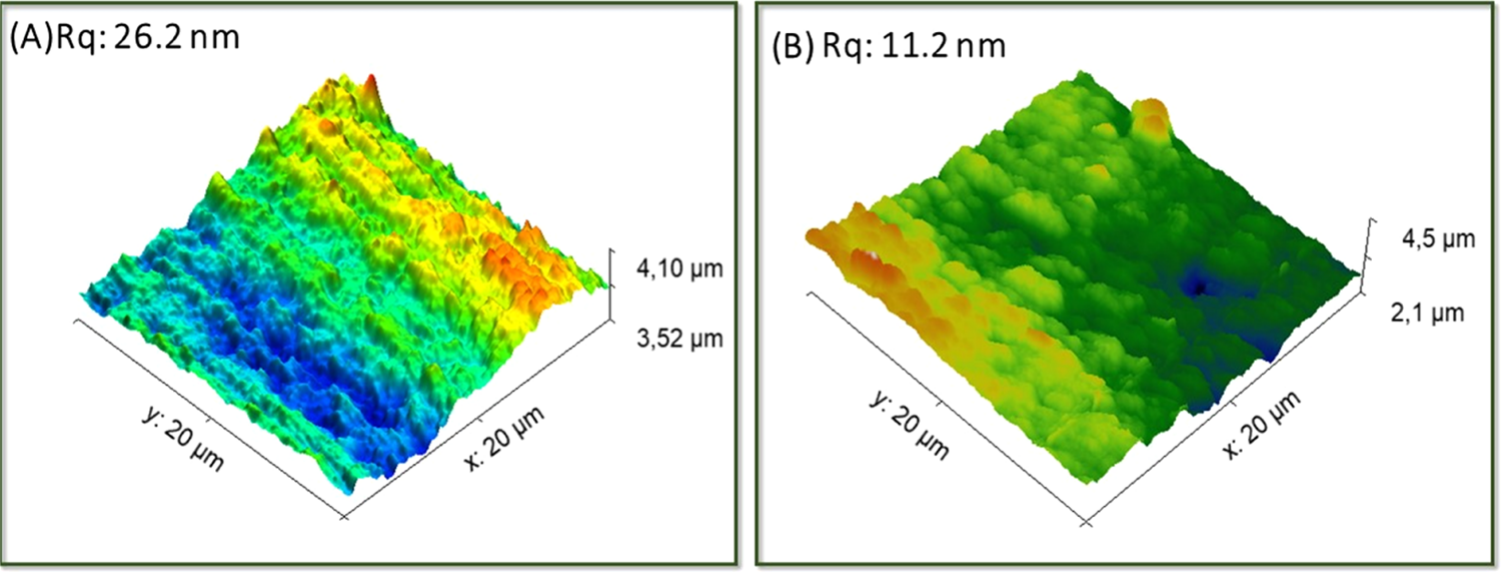
Topography images obtained from the 3D-printed surfaces. (A) Before physical polishing with a root-mean-square (rms) roughness of Rq = 26.2 nm; (B) after physical polishing with a root-mean-square roughness (rms) of Rq = 11.2 nm.

Surface roughness is a fundamental parameter to evaluate the material’s ability to reflect radiation with minimal attenuation of light intensity. A rough surface scatters the incident light in various directions; the higher and lower parts of the surface reflect light differently, resulting in diffuse scattering rather than a direct and organized reflection. This phenomenon is particularly pronounced for shorter wavelengths. In this context, the surface roughness of the 3D-printed substrates was evaluated by atomic force microscopy (AFM) before and after the polishing procedure, and the topography of the surfaces is represented in Figure 3A,B.

By examining the topographies, it can be noted that the physical polishing procedure resulted in a reduction in surface roughness of approximately 130%, yielding a root-mean-square roughness (RMS) of 11.2 nm, which corresponds to the required mirror flat quality <λ//10 for the UV range.

**Figure 4:**
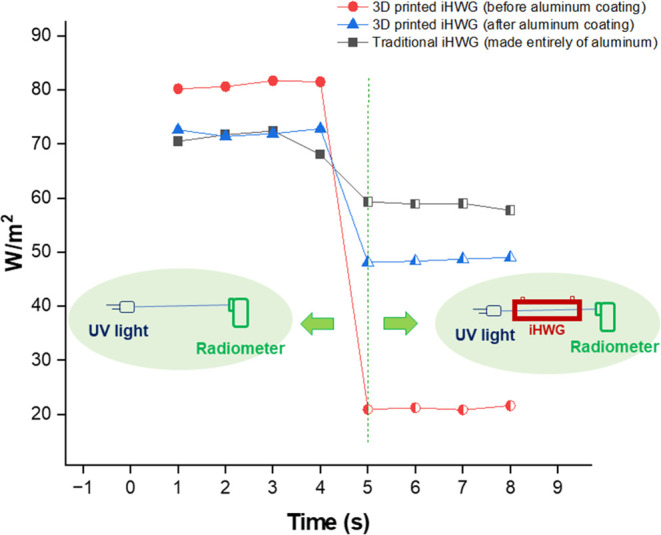
UV irradiation for three iHWGs: Al-iHWG, 3D-printed iHWG without aluminum coating, and 3D-printed iHWG with aluminum coating.

Next, irradiance measurements were performed to evaluate the efficiency of the 3D-printed iHWG in reflecting UV radiation. For this wavelength region, coating surfaces with aluminum is an excellent choice to increase the reflections as it has free electrons in its valence layer that are highly mobile and capable of re-emitting the absorbed UV light. Furthermore, aluminum contributes to the surface smoothness. To assess the reflectivity of the hollow channel, irradiance studies were conducted, specifically targeting the UV region. The experimental setup involved positioning a radiometer at a fixed distance from the radiation source, and measurements were obtained both before and after insertion of the cells between the UV source and the radiometer. A recording frequency of 1 s was employed, with measurements collected for 5 s before and then during propagation of radiation through the iHWG. Three variations of the iHWG were tested, and the results are demonstrated in Figure 4: (i) conventional iHWG fabricated with aluminum; (ii) 3D-printed iHWG without an aluminum coating; and (iii) 3D-printed iHWG with an aluminum film coating.

The propagation of radiation through a miniaturized 3D-printed cell, without the aluminum film coating, had a decrease in light power of 7.5 W/m^2^ for each centimeter of the optical path. On the other hand, when the 3D-printed cell was covered with an aluminum film, the light power loss was reduced to 2.25 W/m^2^, accompanied by a significant improvement in reflectivity of approximately 230%. In addition, radiance tests were performed on conventional Al-iHWGs, showing a light power suppression of approximately 1.56 W/m^2^ for every centimeter of the optical path. Therefore, the aluminum film proved to be suitable for promoting the reflection of UV radiation within the 3D-printed iHWG.

### 3.2 3D-Printed iHWG Coupled to a Portable UV Spectrometer for the Detection of Isoprene and Acetone

The application of the 3D-printed Al-coated iHWG was demonstrated as a proof-of-concept for the determination of the vapors of isoprene and acetone. Isoprene is a highly volatile and toxic substance, and its quantification is also important because it is an indicator of abnormal physiological conditions and can be found at high levels in the breath of patients with lung diseases such as asthma and chronic obstructive pulmonary disease (COPD).^23−25^

The presence of elevated levels of acetone in breath provides valuable insights into glucose metabolism dysregulation, which renders its analysis particularly pertinent in the context of diabetes monitoring and diagnosis. It is a biomarker of importance in facilitating early identification of the disease and enabling effective patient management strategies.^26−30^ Furthermore, acetone is frequently applied as a solvent in diverse industrial sectors, thereby posing an increased risk of occupational exposure.^31−33^ Herein, the analytical parameters for the quantification of both vapors were acquired using the UV-3D-printed Al-coated iHWG sensing device.

**Figure 5:**
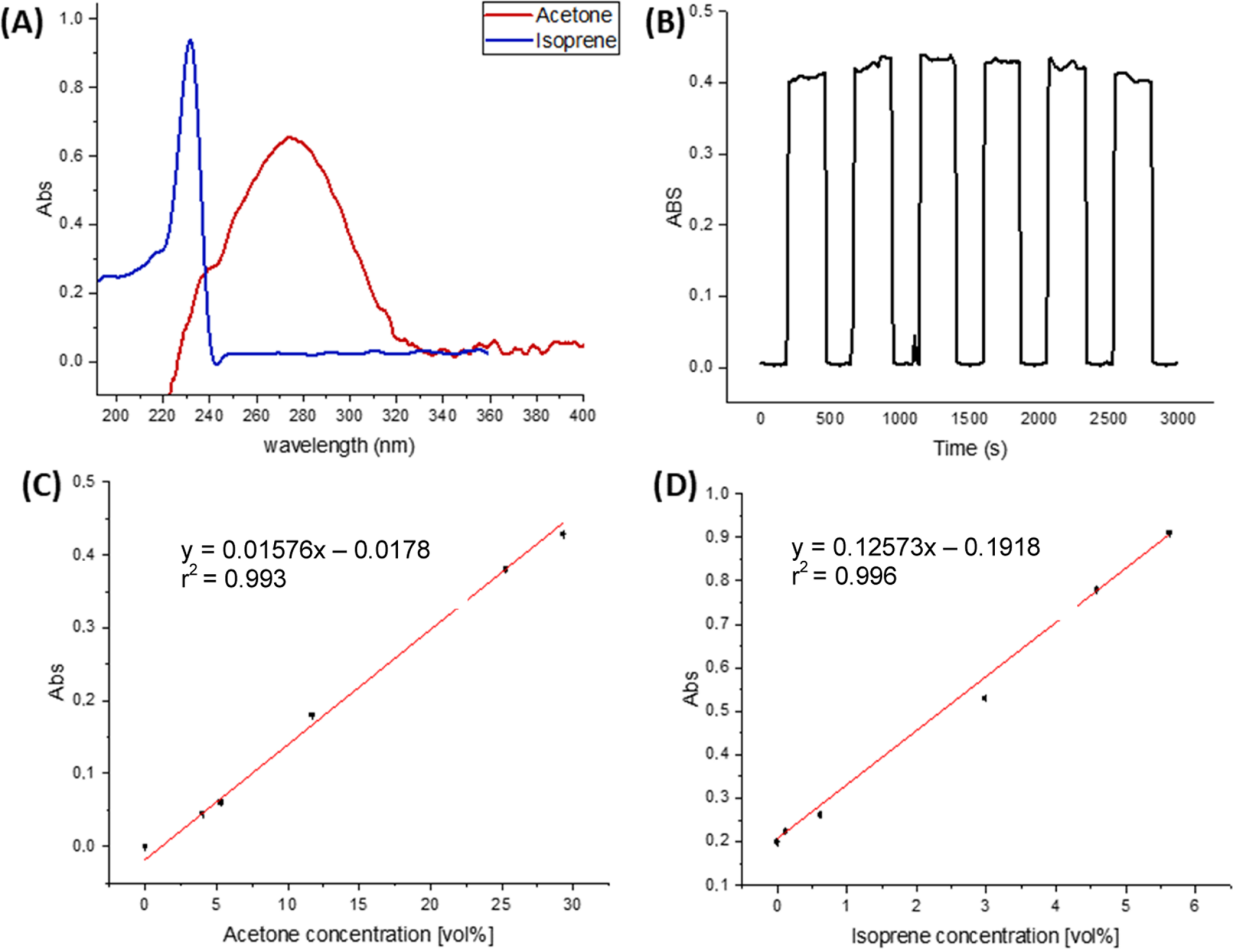
(A) UV spectra of isoprene and acetone; (B) repeatability of successive measurements of acetone vapor; (C) calibration function of acetone at 280 nm; and (D) calibration function of isoprene at 228 nm.

Figure 5a shows the respective UV absorbance signature of isoprene and acetone at different concentrations, with the absorption peak of isoprene at 228 nm (absorption cross-section 8.29 × 10^–18^ cm^2^ mol^–1^)^34^ and at 280 nm for acetone (absorption cross-section 4.77 × 10^–20^ cm^2^ mol^–1^).^35^ Initially, the precision of the analytical response was evaluated by monitoring the analytical signal at 228 nm during 25 independent measurements of 20% of acetone. The achieved intraday relative standard deviation (RSD) was 1.05%. Calibration functions for both analytes were established, enabling quantitative data analysis based on the evaluation of the absorbance vs the isoprene and acetone concentration. For each concentration, the mean value of five replicate measurements was calculated. The sensing system revealed excellent linearity (*R*^2^ > 0.99) over the evaluated concentration range for each vapor, as shown in Figure 5c,d. The limits of detection (LOD) and quantification (LOQ) were considered to be three and ten times the standard deviation of the blank signal, respectively, and were determined at 0.22 and 0.73% for acetone and 0.03 and 0.12% for isoprene. Additionally, the analytical parameters of the UV-3D-printed Al-coated iHWG sensor were compared to results obtained by the UV–Al-iHWG and CG-FID systems. The analytical performance of the proposed method was similar to the conventional GC-based method, with the advantages of portability, lower cost, and response time. Table 2 summarizes the analytical performance of the method.

**Table 2:** Analytical Performance of the Proposed System Compared with the Gas Chromatography Method with Flame Ionization Detection

	GC-FID		Al-iHWG		3D-printed Al-coated iHWG	
	acetone	isoprene	acetone	isoprene	acetone	isoprene
LOD (%)	0.47	0.05	0.42	0.06	0.22	0.03
LOQ (%)	1.55	0.17	1.38	0.21	0.73	0.12
*R*^2^	0.9961	0.9903	0.9939	0.9939	0.993	0.996
linear range (%)	3.30–270.3	0.17–1.69	5.43–15.9	0.21–2.77	4.03–29.32	0.12–5.63
noise (mAU)			0.0006	0.0003	0.0013	0.0027
RSD (%)^a^	7.0	6.8	5.2	4.3	4.9	5.8
sensibility			0.00453	0.16271	0.01576	0.12573
response time (min)	6.3	5.7	0.3	0.3	0.3	0.3

a *n* = 5.

## 4 Conclusions

In this study, we present an SLA 3D-printed miniaturized gas cell for the design of miniaturized sensor systems in the UV spectra range. The iHWG was printed using photocurable resins followed by physical polishing and aluminum coating for postprocessing of the surface, yielding the desired surface quality for efficiently propagating UV radiation. The combination of the 3D-printed gas cell with a UV light source and a portable UV spectrometer was demonstrated during the proof-of-concept quantification of isoprene and acetone in vapor samples. The analytical performance was comparable to conventional analytical methods, including gas chromatography coupled to flame ionization detection. The portability, affordability, and adaptability of the 3D-printed device render it ideally suitable for a wide variety of sensing scenarios, facilitating, e.g., the early detection of physiological abnormalities, real-time environmental monitoring, etc. Furthermore, advancements in optical device technology, including deep UV-LEDs, will further enhance the development of portable and cost-effective optical sensing devices in the ultraviolet spectral regime.
